# Molecular remission at T cell level in patients with rheumatoid arthritis

**DOI:** 10.1038/s41598-021-96300-z

**Published:** 2021-08-17

**Authors:** Jun Inamo, Katsuya Suzuki, Masaru Takeshita, Yasushi Kondo, Yuumi Okuzono, Keiko Koga, Yoshiaki Kassai, Maiko Takiguchi, Rina Kurisu, Akihiko Yoshimura, Tsutomu Takeuchi

**Affiliations:** 1 Division of Rheumatology, Department of Internal Medicine, School of Medicine, Keio University, 35 Shinanomachi, Shinjuku-ku, Tokyo, 160-8582 Japan; 2 Immunology Unit, Takeda Pharmaceutical Co Ltd, Fujisawa-Shi, ResearchKanagawa, Japan; 3 Department of Microbiology and Immunology, School of Medicine, Keio University, Shinjuku-ku, Tokyo, Japan

**Keywords:** Rheumatoid arthritis, Machine learning, Predictive medicine, Transcriptomics

## Abstract

While numerous disease-modifying anti-rheumatic drugs (DMARDs) have brought about a dramatic paradigm shift in the management of rheumatoid arthritis (RA), unmet needs remain, such as the small proportion of patients who achieve drug-free status. The aim of this study was to explore key molecules for remission at the T cell level, which are known to be deeply involved in RA pathogenesis, and investigate the disease course of patients who achieved molecular remission (MR). We enrolled a total of 46 patients with RA and 10 healthy controls (HCs). We performed gene expression profiling and selected remission signature genes in CD4^+^ T cells and CD8^+^ T cells from patients with RA using machine learning methods. In addition, we investigated the benefits of achieving MR on disease control. We identified 9 and 23 genes that were associated with clinical remission in CD4^+^ and CD8^+^ T cells, respectively. Principal component analysis (PCA) demonstrated that their expression profiling was similar to those in HCs. For the remission signature genes in CD4^+^ T cells, the PCA result was reproduced using a validation cohort, indicating the robustness of these genes. A trend toward better disease control was observed during 12 months of follow-up in patients treated with tocilizumab in deep MR compared with those in non-deep MR, although the difference was not significant. The current study will promote our understanding of the molecular mechanisms necessary to achieve deep remission during the management of RA.

## Introduction

Rheumatoid arthritis (RA) is an autoimmune disease characterized by chronic inflammation of the synovial tissue^1^. The advent of disease-modifying anti-rheumatic drugs (DMARDs) has brought about a dramatic paradigm shift in the management of RA. Currently, the goal for RA treatment is to achieve clinical remission (CR), which has been facilitated by the development of various types of biological agents. However, unmet needs in the management of RA remain for a large proportion of patients with remission. More than half of patients with CR experience an RA flare following DMARD cessation^2,3^. Moreover, the existence of patients with difficult-to-treat RA^4^, who ultimately become resistant to multiple types of DMARDs, introduces the need to understand the molecular status of remission.

Thus, molecular remission (MR) has been proposed to distinguish patients with “deep” remission from others^5,6^. A proteome study demonstrated that a low multi-biomarker disease activity score, which the researchers developed, was associated with limited radiographic progression over the following 12 months^5^. We recently investigated molecular signatures that were associated with deep remission at the multi-omics level^6^. In the previous report, drug treatments altered the molecular profile to better resemble that of healthy controls (HCs) at the transcriptomic, serum proteomic, and immunophenotypic levels. In addition, longitudinal monitoring suggested that the achievement of MR by DMARDs was associated with long-term stable CR. However, how each transcriptomic remission signature molecule is related to clinical traits remained unclear because we used whole-blood specimens, and the expression profiles varied according to cell subsets, indicating the necessity of further study using each cell subpopulation^6^.

T cells are well-known to contribute to the pathogenesis of RA. Susceptible genes for RA outside the major histocompatibility complex locus are highly expressed in CD4^+^ T cells^7,8^. The evidence that a subset of CD8^+^ T cells is also critical a contributor to the development of RA is accumulating. CD8^+^ T cells are required for the development of ectopic germinal centers in the synovium, which is considered to be the home of the core immune response in RA^9^. Recently, to investigate and clarify the comprehensive characteristics of T cells in RA, we conducted a multi-dimensional, immunophenotyping analysis according to the developmental stage: CD4^+^ T cells were classified into four subsets, naïve (TN), stem cell memory (TSCM), central memory (TCM), and effector memory (TEM), whereas CD8^+^ T cells were classified into five stages, TN, TSCM, TCM, TEM, and CD45RA-positive effector memory (TEMRA). The study demonstrated that the CD8^+^ TEMRA subset increased in patients with RA compared with HCs, and TEM-follicular helper (Tfh) cells and TEM-T helper 17(Th17) cells were correlated with disease activity, suggesting that T cells in patients with remission may represent the MR state of RA^10^.

Here, we report the key molecules associated with remission at the T cell level and investigate the disease course of patients who achieved MR.

## Methods

### Patients and control subjects

**Figure 1:**
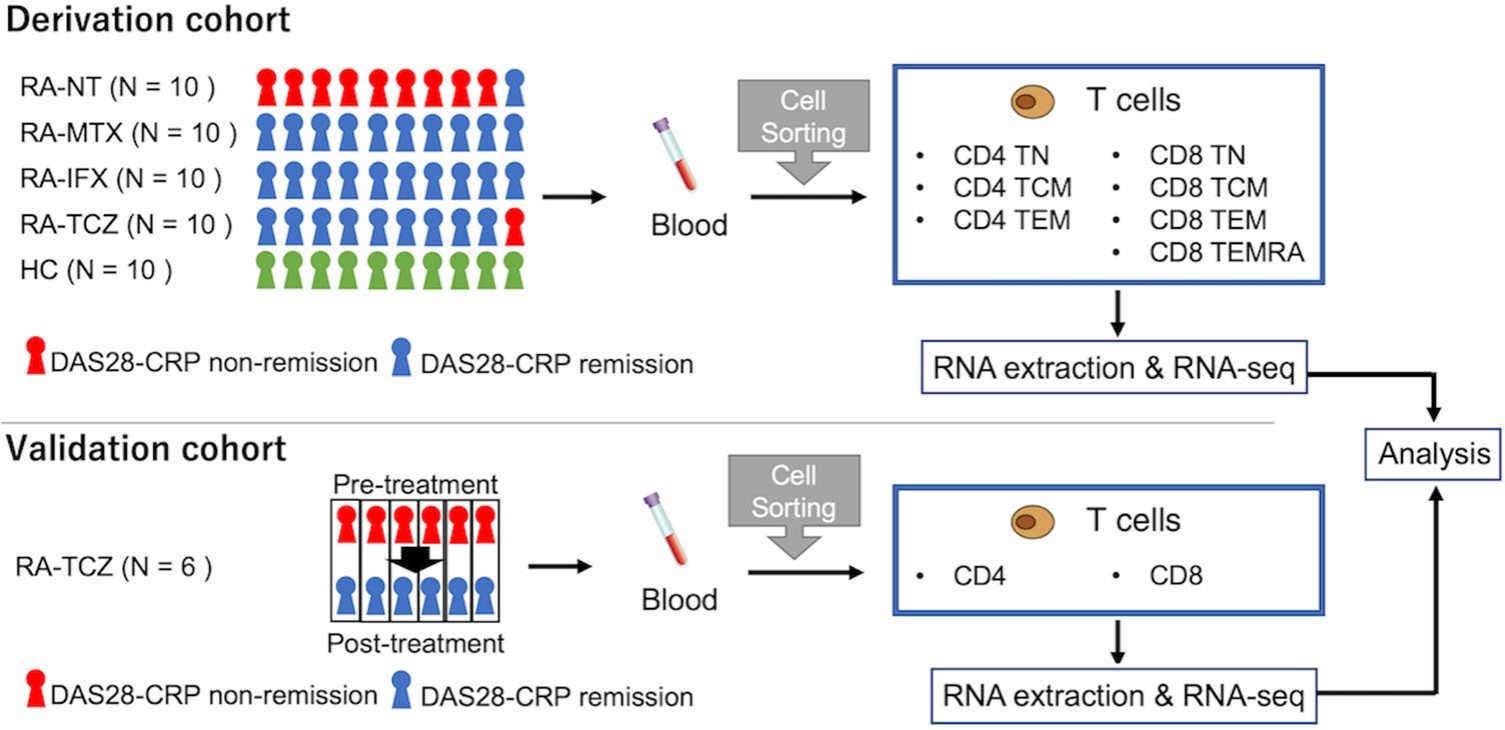
Workflow of the current study. We used 2 cohorts: a derivation cohort including cross-sectional subjects and a validation cohort including longitudinal subjects treated by TCZ. In the derivation cohort, we measured gene expression according to cell subpopulations and summarized all CD4^+^ T cells and CD8^+^ T cells in the expression analysis. HC, healthy control; IFX, infliximab; MTX, methotrexate; RA, rheumatoid arthritis; NT, non-treatment; TCM, central memory T cell; TCZ, tocilizumab; TEM, effector memory T cell; TEMRA, CD45RA-positive effector memory T cell; TN, naïve T cell.

The current study utilized cohorts from our previous report^10^. The detailed information was described previously. Briefly, 2 cohorts were included in this study (Cohort 6 and Cohort 7 of the previous report, Supplementary Table S1-2). Cross-sectional gene expression profiling was performed in the derivation cohort, and longitudinal gene expression profiling was performed at pre-treatment and post-treatment time points in the validation cohort (Fig. 1). Peripheral blood samples and synovial fluid (SF) samples were collected before treatment from patients with drug-naïve RA and during treatment from other patients, respectively. We separated each T cell subpopulation from the specimens, and RNA was isolated. Then, we conducted RNA-sequencing and performed bioinformatic analysis. Read count normalization was performed using the transcripts per million (TPM) method^13^. Disease activity was assessed by standard composite indices, such as DAS28-ESR (disease activity score 28-ESR) and DAS28-CRP, and remission was defined as a DAS28-CRP < 2.4^14^. Five of six patients treated by tocilizumab in the validation cohort achieved Clinical Disease Activity Index (CDAI) remission and one patient achieved low disease activity (CDAI 2.9) (Supplementary Table S1). This study was approved by the Institutional Review Board of Keio University School of Medicine and conducted according to the Declaration of Helsinki. Consent to participate was obtained from all subjects in the current study before blood specimen was collected.

### Development of RA remission signature models

To select genes that had a strong relationship with the remission, we applied the least absolute shrinkage and selection operator (lasso) to normalized gene expression data of patients with drug-naïve RA and treatment in the derivation cohort. Lasso is a machine learning method that is suitable for detecting key variables without prior feature selection from a multivariate dataset that contains only a few covariates that are associated with the outcome, which improves the prediction accuracy and interpretability of regression models^15^. To avoid overfitting, we first divided the gene expression dataset of the derivation cohort into a training dataset and a test dataset (with ratio = 7:3) and conducted lasso using the training dataset with seven-fold cross-validation. Then, a partial least-squares regression (PLS-R) was utilized to weight the values of selected genes and construct a model that was useful for separating remission and non-remission patients. The variable importance in projection (VIP) score obtained by PLS-R is a significant measurement for each predictor variable. Genes with VIP scores greater than 1 were considered to be related to remission^16^. Thus, we can evaluate the importance of each gene. Receiver operating characteristic (ROC) analysis was used to evaluate the prediction accuracy of the test data using the statistical model generated from the training set. Then, the statistical model was applied to the whole dataset to enumerate the “remission odds” for each sample. The caret and pls R package were used for lasso and PLS-R modeling, respectively^17^.

### Principal component analysis and pathway mapping

Principal component analysis (PCA) was performed using normalized data with the FactoMineR R package^18^. To gain a functional annotation of selected genes, Enrichr’s plugin^19^ KEGG pathways^20^ was used.

### Statistics

Continuous data are presented as the median and interquartile range or as a number and percentage, as appropriate. The Wilcoxon rank-sum test was used to examine differences between continuous variables. Fisher’s exact test was used to compare proportions in categorical data between groups. All statistical analyses were performed with R (R Foundation for Statistical Computing, Vienna, Austria).

## Results

### Remission signature genes in RA

The primary objective was to explore genes that are relevant to remission status in RA (referred to as remission signature genes). Therefore, we first selected genes that were useful for separating patients with remission from those with non-remission using a training dataset from the derivation cohort, separated according to CD4^+^ and CD8^+^ T cells. Using lasso on all 15,304 transcripts, 17 and 46 genes were selected as important molecules in CD4^+^ and CD8^+^ T cells, respectively, to classify patients with remission from those with non-remission.

**Figure 2:**
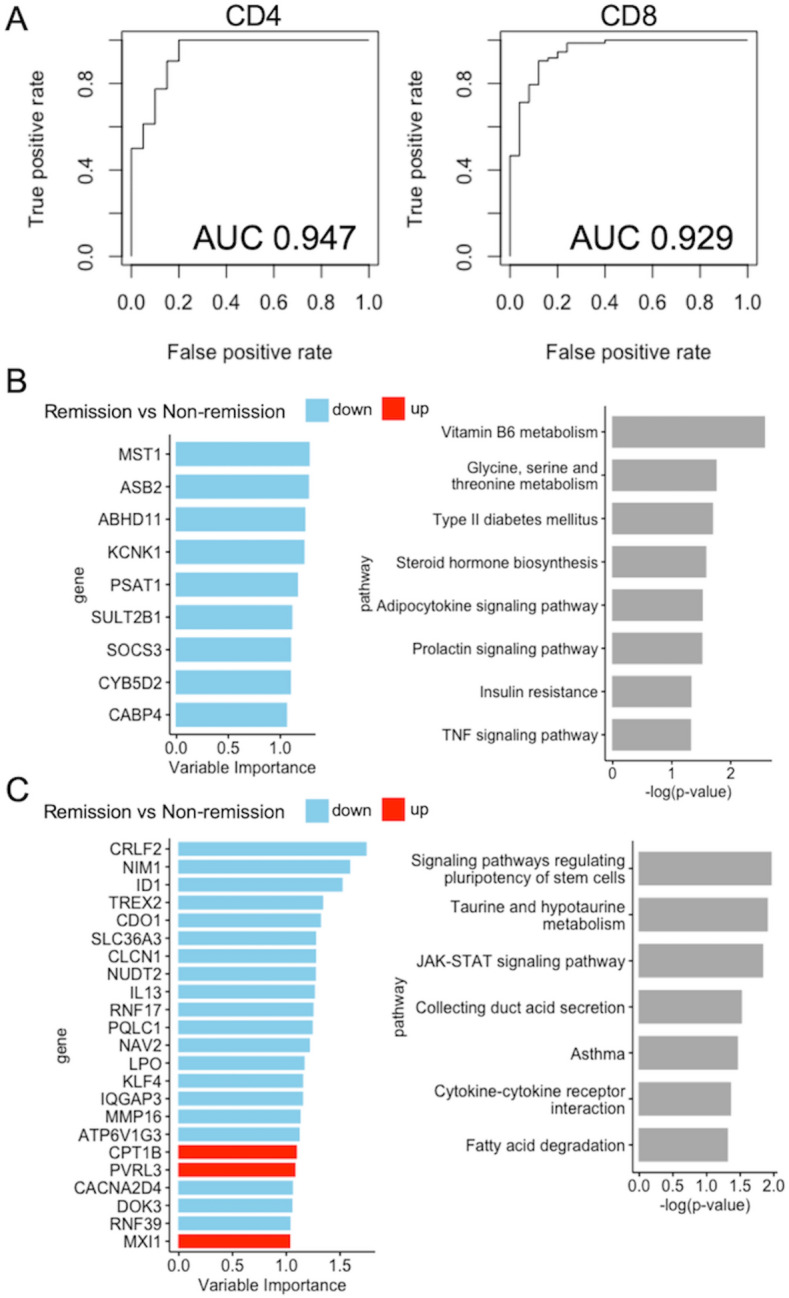
Identification of molecular signatures associated with remission in RA. (**A**) ROC analysis according to the statistical model generated by the combination of lasso and PLS-R. (**B**) and (**C**) Remission signature genes (left) and enrichment analysis (right) of CD4^+^ T cells and CD8^+^ T cells (**B**). The PLS-R analysis of gene expression, with blue indicating low and red toward high expression in remission patients. AUC, area under the curve; PLS-R, a partial least-squares regression; RA, rheumatoid arthritis; ROC, receiver operating characteristic.

Then, we weighted the genes selected by lasso and constructed a statistical model to separate remission from non-remission (referred to as the RA remission signature model) by applying PLS-R. The ROC analysis applied to the test set that was not used for training was separated able to separate the two populations with good accuracy (area under the curve [AUC], 0.947 and 0.929 for CD4^+^ and CD8^+^ T cells, respectively; Fig. 2A). This result indicated that the combination of lasso and PLS-R captured informative genes from our data. In addition, 9 (e.g., *MST1, ASB2, SULT2B1,* and *SOCS3*) and 23 (e.g., *CRLF2, NIM1,* and *ID1*) genes met the criteria (VIP > 1) for model inclusion in CD4^+^ and CD8^+^ T cells, respectively (Fig. 2B,C and Supplementary Table S3). Hereafter, we refer to these genes as remission signature genes. To understand the function of remission signature genes, pathway analysis was performed (Fig. 2B,C). In CD4^+^ T cells, molecules involved in various metabolic pathways (Vitamin B6 metabolism and Glycine, serine, and threonine metabolism), endocrine pathways (steroid hormone biosynthesis, adipocytokine signaling pathway, prolactin signaling pathway, and insulin resistance), and the TNF signaling pathway were enriched. In CD8^+^ T cells, molecules involved in metabolic pathways (taurine and hypotaurine metabolism and fatty acid degradation) and the JAK-STAT signaling pathway were enriched.

### Remission odds of each T cell subpopulation

To investigate the effects of different DMARDs and on remission signature genes in the various T cell subpopulations (TN, TCM, TEM, and TEMRA), we compared remission odds according to each subgroup. Using the RA remission signature model generated by the combination of lasso and PLS-R, the remission odds of each subject were produced: if patients were in remission status, remission odds were > 0.5, whereas if patients were far from remission, remission odds were < 0.5. Because T cells in SF are considered to reflect pathological status, we calculated the remission odds of the T cell subpopulation in SF from some patients.

Evaluation of model goodness of fit and quality of prediction by cross-validation showed that the model for CD4^+^ T cells performed better than the model for CD8^+^ T cells; the model for CD4^+^ T cells explained a higher variance (65.7%) of the response variable of remission or non-remission, and the root mean square error of prediction was minimal when one component, while the model for CD8^+^ T cells explained only less than 25% of the variance in the response variable (Supplementary Table S4).

**Figure 3:**
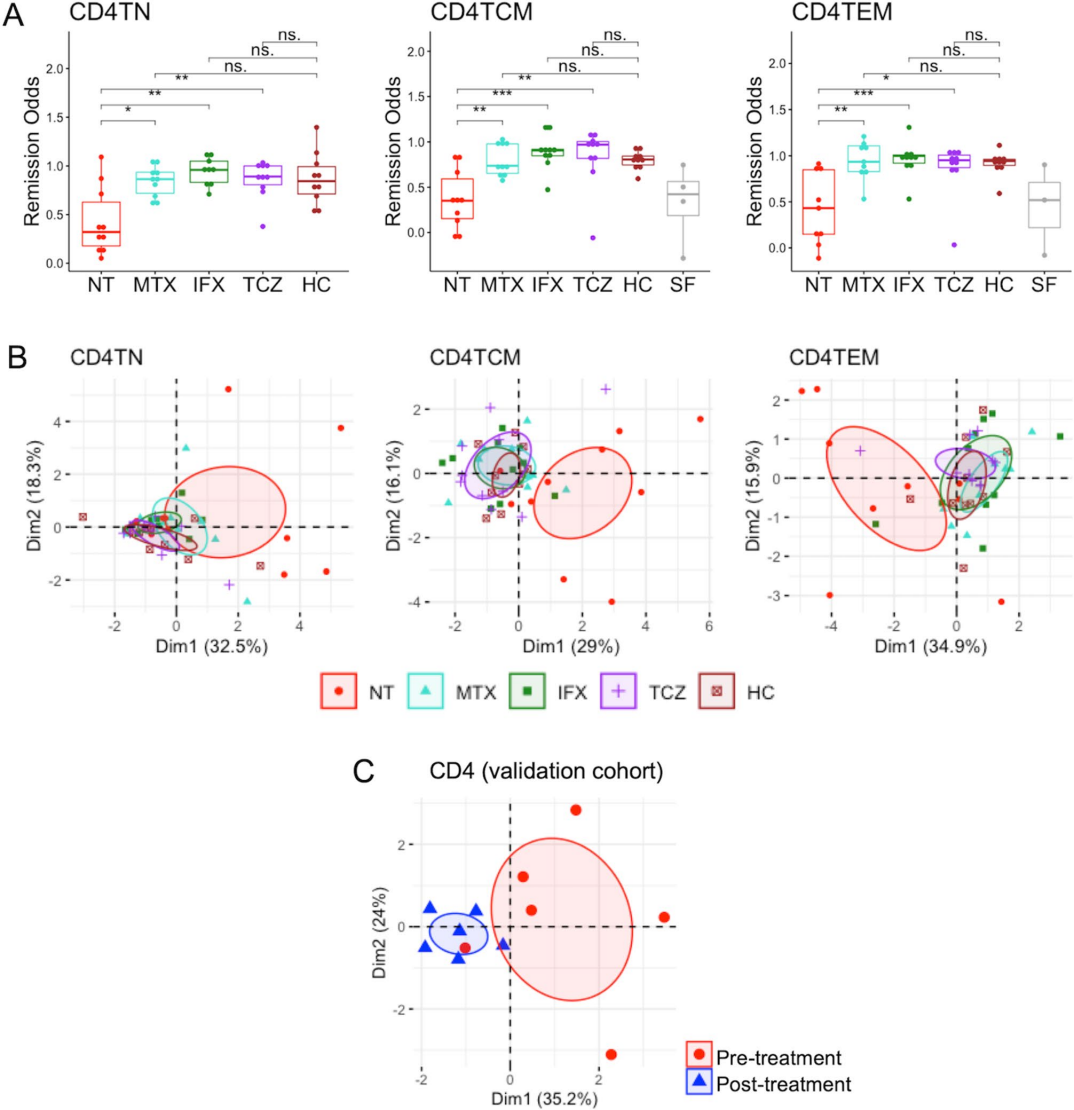
Remission odds and gene expression profiling of remission signature genes in CD4^+^ T cells. (**A**) Remission odds generated by the RA remission model. ‘*’: *p* < 0.05, ‘**’: *p* < 0.01, ‘***’: *p* < 0.001. (**B**) and (**C**) Principal component analysis using remission signature genes in the derivation cohort (**B**) and validation cohort (**C**). The ellipse shows the 95% confidence interval of the value of the principal component analysis. Patients in the validation cohort were treated by TCZ for 6 months and achieved remission after treatment. HC, healthy control; IFX, infliximab; MTX, methotrexate; RA, rheumatoid arthritis; NT, non-treatment; SF, synovial fluid; TCM, central memory T cell; TCZ, tocilizumab; TEM, effector memory T cell; TN, naïve T cell.

In CD4^+^ T cells, the remission odds of patients with DMARDs, all of whom were in remission, except for one patient (Fig. 1), were significantly higher compared with those of drug-naïve patients (Fig. 3A). Of note, although significance was not achieved due to limited samples, SF samples trended toward low remission odds, as did drug-naïve samples from peripheral blood, suggesting that remission signature genes might represent the pathogenic status of RA. Compared with HCs, the DMARD-treated patients had similar values, regardless of drug type, indicating that all drugs pushed the pathogenic gene expression profile of remission signature genes toward the healthy state. Correspondingly, PCA using remission signature genes in CD4^+^ T cells demonstrated that only drug-naïve samples clustered from the other groups (Fig. 3B). To validate the classification ability of remission signature genes, PCA analysis was conducted using expression data from the validation cohort. Similar to earlier results, samples from patients in remission created clusters apart from those in non-remission, supporting the robustness of remission signature genes in CD4^+^ T cells (Fig. 3C).

**Figure 4:**
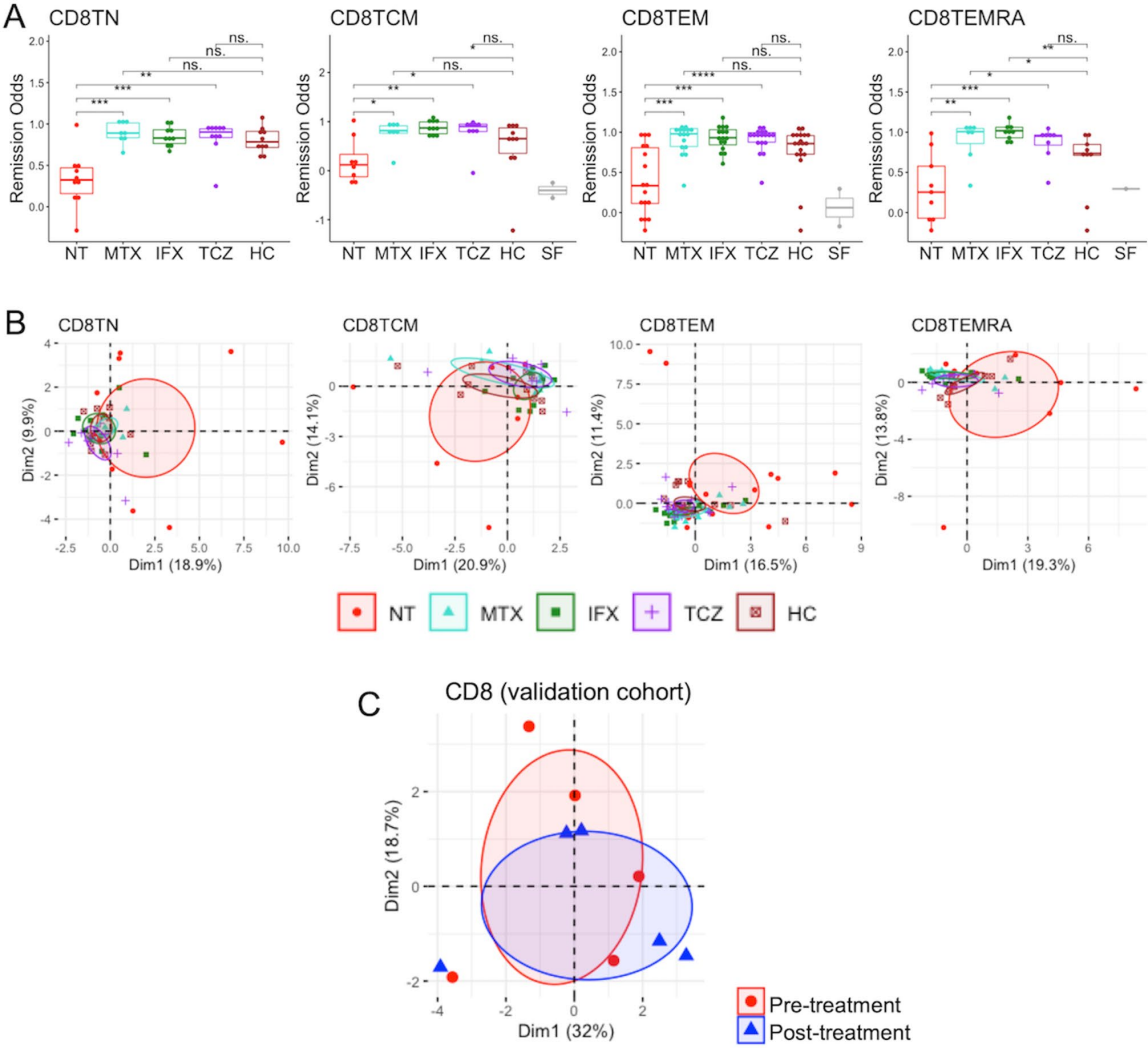
Remission odds and gene expression profiling of remission signature genes in CD8^+^ T cells. (**A**) Remission odds generated by the RA remission model. ‘*’: *p* < 0.05, ‘**’: *p* < 0.01, ‘***’: *p* < 0.001, ‘****’: *p* < 0.0001. (**B**) and (**C**) Primary component analysis using remission signature genes in the derivation cohort (**B**) and the validation cohort (**C**). The ellipse shows the 95% confidence interval of the value of principal component analysis. Patients in the validation cohort were treated by TCZ for 6 months and achieved remission after treatment. HC, healthy control; IFX, infliximab; MTX, methotrexate; RA, rheumatoid arthritis; NT, non-treatment; SF, synovial fluid; TCM, central memory T cell; TCZ, tocilizumab; TEM, effector memory T cell; TEMRA, CD45RA-positive effector memory T cell; TN, naïve T cell.

In the CD8^+^ T cell subpopulation, similar to CD4^+^ T cells, all remission odds of patients with DMARDs were significantly higher than those of drug-naïve patients (Fig. 4A). However, the remission odds of some samples in groups of DMARDs were also significantly higher than those of HCs, suggesting that selected genes in CD8^+^ T cells might not correctly represent the healthy state. In addition, PCA demonstrated that all clusters overlapped, except the TEM subpopulation (Fig. 4B). Further, PCA using validation cohort was unable to validate the selected genes, indicating the vulnerable ability of remission signature genes in CD8^+^ T cells (Fig. 4C).

### Relations between molecular remission and following disease activity

**Figure 5:**
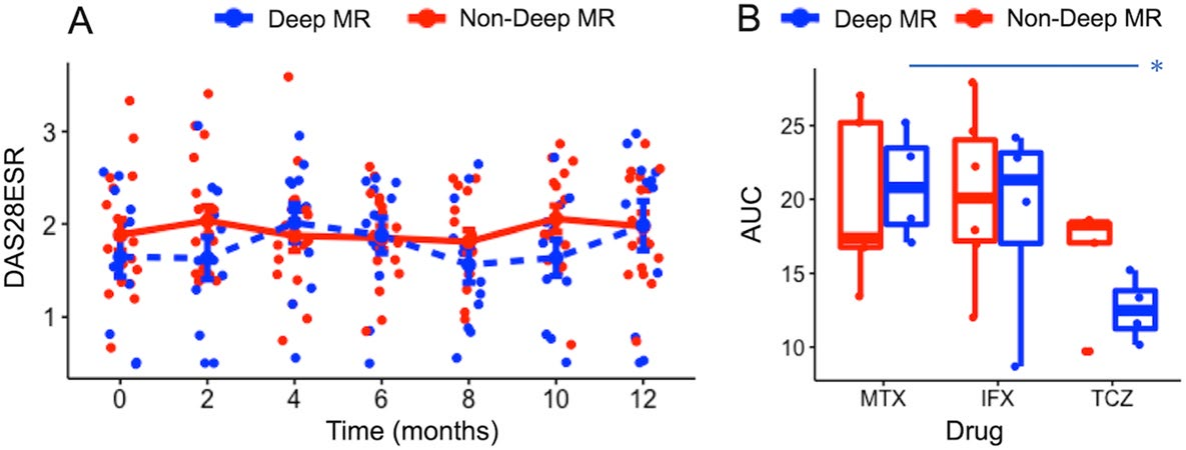
Relationship between MR and disease activity. (**A**) The changes in DAS28-ESR during the follow-up period, which was split into two-week intervals. For each group, the average ± standard error of DAS28-ESR within the same interval was shown by the line. (**B**) Comparison of AUC (commutative DAS28-ESR during the 12-month follow-up) according to drug type. ‘*’: *p* < 0.05. AUC, area under the curve; DAS28-ESR, disease activity score-28 ESR; IFX, infliximab; MTX, methotrexate; TCZ, tocilizumab.

To elucidate the benefits of MR, we next addressed whether any differences existed between patients in “deep” MR and non-deep MR. To achieve this goal, we conducted a follow-up study of 29 consecutive patients (MTX, n = 10; IFX, n = 10; TCZ, n = 9) treated with DMARDs in the derivation cohort for up to 12 months after the measurement of gene expression. We defined the MR of each cell subset as remission odds greater than the average value of the remission odds for each cell subset, and deep MR was defined as each patient with greater than 4 cell subsets in MR (maximum 7). Of the 29 patients treated with DMARDs, 12 and 17 patients were classified as deep MR and non-deep MR. Disease activity was not significantly different at any time point (Fig. 5A). However, the cumulative DAS28-ESR (described as the AUC) of patients treated with TCZ in deep MR had a trend lower than those treated with TCZ in non-deep MR (12.48 [11.25–13.82] vs. 18.26 [17.07–18.36], p = 0.19; Fig. 5B). Comparisons among drug types showed a significant difference between patients treated with TCZ and those treated with MTX in deep MR. Although we conducted sensitivity analysis by changing the outcome (e.g., DAS28-CRP and each component of the DAS28) and the definition of deep MR (e.g., the cut-off number of the cell subpopulation, limited to the CD4^+^ T cell subpopulations), we could not find significant benefit for deep MR in our data (data not shown).

## Discussion

In the current study, we identified remission signature genes associated with RA in CD4^+^ and CD8^+^ T cells. Although those in CD8^+^ T cells were vulnerable when applied to another cohort, those in CD4^+^ T cells had the robust ability to classify remission and non-remission patients in both cross-sectional and longitudinal cohorts. Furthermore, the remission odds calculated by the RA remission model in CD4^+^ T cells showed similar value among patients in remission and HCs, suggesting that the 9 selected genes represent whether the CD4^+^ T cells of RA are pushed back to a healthy state. In addition, deep MR by TCZ had the potential to be associated with better disease control.

As shown in previous reports, T cells were involved in the development and chronicity of RA^7–10^. Compared with CD4^+^ T cells, evidence supporting a role for CD8^+^ T cells in RA is still emerging^9^. Clonal expansion was observed for CD8^+^ T cells but not for CD4^+^ T cells in newly diagnosed patients with RA, indicating that CD8^+^ T cells are necessary for the initial phase of RA^21^. However, RA risk alleles were preferentially expressed in CD4^+^ T cells but not in CD8^+^ T cells^7,8^, and only the CD4^+^ T cell subpopulation was positively associated with disease activity^10^, suggesting that CD4^+^ T cells more deeply contribute to the activity state of RA than CD8^+^ T cells.

Remission signature genes identified in CD4^+^ T cells were downregulated in patients with remission. *MST1* promotes the migration of T cells via the activation of LFA-1^22,23^. In addition, *MST1*-deficient T cells are prone to apoptosis^24^. *ASB2* is known to promote NF-κB activation, leading to the suppression of T cell apoptosis^25^. *SULT2B1* is involved in cholesterol homeostasis and is expressed in activated T cells, prompting proliferation via the inhibition of LXR signaling^26^. *SOCS3* inhibits STAT3, a downstream molecule in the JAK-STAT pathway that is inhibited by TNFα- and IL-6 inhibition^27^; therefore, the downregulation of *SOCS3* may lead to the activation of the inflammatory pathway^28^. However, the loss of *SOCS3* in CD4^+^ T cells promotes anti-inflammatory cytokines, such as interleukin 10 and transforming growth factor-beta 1, and suppresses inflammatory responses^29^, suggesting that the decreased expression of *SOCS3* itself may be beneficial for controlling RA. Among the other 5 genes identified as remission signature genes in CD4^+^ T cells (*ABHD11, KCNK1, PSAT1, CYB5D2,* and *CABP4*), their functions in T cells remain unknown, and further functional study is needed to clarify their significance in RA.

However, we were unable to show any significant benefit for clinical course associated with achieving deep MR, as defined by remission signature genes, although those treated with TCZ in deep MR had a favorable trend. To date, although several studies have attempted to predict changes in disease activity using only transcriptome data, they have failed to show robust predictability^6,30^. In our data, most patients, even non-deep MR patients, were under good control during follow-up, as shown in Fig. 5A, which might make the detection of significant differences challenging. In addition, the definition of “deep” MR used in the current study lacks supporting evidence. Therefore, to explore the impact of MR on clinical traits and the associations between MR and drug types, we need to plan a prospective study with larger sample size.

This study suffers from several limitations. First, we did not examine the association between functional subpopulations and MR. We previously reported that Tfh and Th17 cells in TEM were correlated with disease activity^10^. In addition, the proportion of T peripheral helper cells, which act as pathogenic CD4 helper T cells in RA, is associated with disease activity and treatment^31^. These findings suggest that the extent of T cell contributions to disease status varies and indicates the necessity of exploring remission signature genes according to their functions^10^. Second, the functions of these genes in T cells have not been investigated. In vitro or in vivo studies remain necessary to determine whether these genes are potential targets for therapy. Third, other potential benefits of MR were not considered in the current study, such as a lower risk of flares. Forth, the remission signature genes identified in this study might be affected by the confounding factors, such as drug effects and disease activity, because patients with established RA on therapy and with active disease were not included. Because the concept of MR in RA has only emerged recently, we need to validate the definition and effects of MR on real-world patients with both active, early RA and established RA in future research using larger cohorts.

## Conclusions

We identified robust remission signature genes in CD4^+^ T cells. The current study will highlight the utility of using transcriptome data in CD4^+^ T cells to classify remission and non-remission in RA and promote the development of novel therapeutic targets against RA.

### Ethics approval and consent to participate

Ethics approval was obtained from the Institutional Review Board of Keio University School of Medicine. Consent to participate was obtained from all subjects in the current study before blood specimen was collected.

## Data availability

Transcriptome data are available at the GEO database. The accession codes are GSE113156 and GSE118829. All custom computer codes in the generation or processing of the described data are available upon reasonable request.

## Supplementary Information

Supplementary Information 1.

Supplementary Information 2.

Supplementary Information 3.

## Abbreviations

AUC: Area under the curve
TEMRA: CD45RA-positive effector memory T cells
TCM: Central memory T cells
CR: Clinical remission
DAS28-CRP: Disease activity score-28 CRP
DAS28-ESR: Disease activity score-28 ESR
DMARDs: Disease-modifying anti-rheumatic drugs
TEM: Effector memory T cells
HCs: Healthy controls
IFX: Infliximab
lasso: Least absolute shrinkage and selection operator
MTX: Methotrexate
MR: Molecular remission
TN: Naïve T cells
PLS-R: Partial least-squares regression
PCA: Principal component analysis
ROC: Receiver operating characteristics
RA: Rheumatoid arthritis
TSCM: Stem cell memory T cells
SF: Synovial fluid
Th17: T helper 17 cell
Tfh: T-follicular helper cells
TCZ: Tocilizumab
TPM: Transcripts per million
VIP: Variable importance in projection
