# Supplementary material for: Molecular remission at T cell level in patients with rheumatoid arthritis
Source: Sci Rep. 2021 Aug 17;11:16691. doi: 10.1038/s41598-021-96300-z (PMC8371080; doi:10.1038/s41598-021-96300-z)
Supplement: Supplementary file 1 — Supplementary Information 1. [file 41598_2021_96300_MOESM1_ESM.docx]

| RA (*n*=6) | pre-treat | post-treat |
| --- | --- | --- |
| Age, years | 65 (52-67) |  |
| Female, *n* (%) | 4 (66.7) |  |
| Disease duration, months | 67 (14-293) |  |
| RF positive, *n* (%) | 4 (66.7) |  |
| ACPA positive, *n* (%) | 4 (66.7) |  |
| DMARD use, *n* (%) | 5 (86) |  |
| DAS28-ESR | 4.7 (4.2-5.2) | 0.8 (0.5-1.3) |
| DAS28-CRP | 3.9 (3.5-5.0) | 1.2 (1.0-1.5) |
| SDAI | 15.6 (9.7-26.7) | 1.7 (0.4-2.9) |
| CDAI | 14.7 (8.0-25.7) | 1.7 (0.4-2.9) |
| SJC, 28 joints, *n* | 5 (3-8) | 0 (0-0) |
| TJC, 28 joints, *n* | 5 (3-8) | 0 (0-0) |
| PtGA, mm | 39 (11.5-58.8) | 10 (3.8-20.5) |
| PhGA, mm | 24 (8.8-43.8) | 0 (0-7.5) |
| CRP, mg/dl | 0.9 (0.5-2.0) | 0.0 (0.0-0.0) |
| ESR, mm/h | 41 (13-62) | 2 (2-4) |
| MMP-3, ng/ml | 109 (78-178) | 51 (17-84) |
| HAQ-DI | 1 (1-1.3) | 0.5 (0.1-1.0) |

Supplementary Table S1. Patient backgrounds of cohort 6. [Takeshita M, et al. Ann Rheum Dis. 2019;78:1346-1356.]

RA, rheumatoid arthritis.

|  | HC (*n*=10) | NT (*n*=10) | MTX (*n*=10) | IFX (*n*=10) | TCZ (*n*=10) | SF *** (*n*=4) |
| --- | --- | --- | --- | --- | --- | --- |
| Age, years * | 56 (52-73) | 56 (48-66) | 58 (43-70) | 58 (55-65) | 61 (52-67) | 55 (31-70) |
| Female, *n* (%) * | 8 (80) | 8 (80) | 9 (90) | 9 (90) | 10 (100) | 3 (75) |
| Disease duration, months |  | 5 (2-27) | 104 (52-150) | 72 (57-180) | 120 (66-285) | 92 (12-169) |
| RF positive, *n* (%) |  | 10 (100) | 7 (70) | 8 (80) | 7 (70) | 2 (50) |
| ACPA positive, *n* (%) |  | 10 (100) | 7 (70) | 8 (100) ****** | 7 (70) | 1 (25) |
| DAS28-ESR |  | 4.6 (3.7-5.2) | 2.0 (1.5-2.5) | 1.9 (1.5-2.4) | 1.5 (0.7-1.7) | 2.7 (1.6-5.5) |
| DAS28-CRP |  | 3.9 (3.1-4.2) | 1.3 (1.1-1.5) | 1.2 (1.2-1.2) | 1.2 (1.0-1.4) | 2.5 (2.1-4.8) |
| SDAI |  | 16 (8-21) | 0.8 (0.1-2.7) | 0.5 (0.1-1.0) | 1.0 (0.4-3.0) | 9.4 (6.7-32) |
| CDAI |  | 15 (7-19) | 0.7 (0.0-2.7) | 0.4 (0.0-1.0) | 1.0 (0.4-3.0) | 9.4 (4.2-19.4) |
| SJC, 28 joints, *n* |  | 3 (2-6) | 0 (0-0) | 0 (0-0) | 0 (0-0) | 0.5 (0.0-3.3) |
| TJC, 28 joints, *n* |  | 3 (1-4) | 0 (0-0) | 0 (0-0) | 0 (0-0) | 0 (0-3) |
| PtGA, mm |  | 36 (10-62) | 3 (0-20) | 3 (0-8) | 5 (0-18) | 72 (30-81) |
| PhGA, mm |  | 36 (19-47) | 0 (0-2) | 1 (0-2) | 2 (1-4) | 18 (7.2-54) |
| CRP, mg/dl |  | 0.7 (0.3-1.7) | 0.0 (0.0-0.1) | 0.1 (0.0-0.1) | 0.0 (0.0-0.0) | 1.7 (0.0-13) |
| ESR, mm/h |  | 35 (18-45) | 12 (8-23) | 15 (8-26) | 7 (2-8) | 50 (2-126) |
| MMP-3, ng/ml |  | 62 (47-146) | 25 (16-43) | 24 (17-32) | 31 (20-51) | 284 (78-555) |
| HAQ-DI |  | 0.6 (0.1-1.0) | 0.0 (0.0-0.1) | 0.0 (0.0-0.8) | 0.3 (0.0-0.7) | 1.3 (0.6-2.0) |

Supplementary Table S2. Patient backgrounds of cohort 7. [Takeshita M, et al. Ann Rheum Dis. 2019;78:1346-1356.]

*not significant, Wilcoxon signed-rank test or chi squared test.

***n*=8

*** Three patients were receiving treatment (1 patient receiving MTX, 2 patients receiving MTX and TCZ)

HC, healthy control; IFX, infliximab; MTX, methotrexate; NT, non-treatment; SF, synovial fluid; TCZ, tocilizumab.
