# Supplementary material for: Molecular remission at T cell level in patients with rheumatoid arthritis
Source: Sci Rep. 2021 Aug 17;11:16691. doi: 10.1038/s41598-021-96300-z (PMC8371080; doi:10.1038/s41598-021-96300-z)
Supplement: Supplementary file 3 — Supplementary Information 3. [file 41598_2021_96300_MOESM3_ESM.docx]

|  | RMSEP* | | |
| --- | --- | --- | --- |
|  | (Intercept) | 1 comp | 2 comps |
| CD4^+^ T cells | 0.4347 | 0.2893 | 0.3197 |
| CD8^+^ T cells | 0.4404 | 0.4167 | 0.3947 |

|  | explained variance (%) of response variable** | |
| --- | --- | --- |
|  | 1 comp | 2 comps |
| CD4^+^ T cells | 62.08 | 65.74 |
| CD8^+^ T cells | 12.98 | 23.45 |

Supplementary Table S4. The quality metrics of PLS-R models.

*, Root Mean Square Error of Prediction derived from leave-one-out cross-validated predictions using 10 random segments; **, remission or non-remission. The PLS-R models were fitted by two components.
